# DDX41 Recognizes RNA/DNA Retroviral Reverse Transcripts and Is Critical for *In Vivo* Control of Murine Leukemia Virus Infection

**DOI:** 10.1128/mBio.00923-18

**Published:** 2018-06-05

**Authors:** Spyridon Stavrou, Alexya N. Aguilera, Kristin Blouch, Susan R. Ross

**Affiliations:** a Department of Microbiology and Immunology, College of Medicine, University of Illinois at Chicago, Chicago, Illinois, USA; b Department of Microbiology, Perelman School of Medicine, University of Pennsylvania, Philadelphia, Pennsylvania, USA; Columbia University

**Keywords:** AML/MDS, DEAD-box helicase, antiviral interferon response, cGAS, cytosolic sensing

## Abstract

Host recognition of viral nucleic acids generated during infection leads to the activation of innate immune responses essential for early control of virus. Retrovirus reverse transcription creates numerous potential ligands for cytosolic host sensors that recognize foreign nucleic acids, including single-stranded RNA (ssRNA), RNA/DNA hybrids, and double-stranded DNA (dsDNA). We and others recently showed that the sensors cyclic GMP-AMP synthase (cGAS), DEAD-box helicase 41 (DDX41), and members of the Aim2-like receptor (ALR) family participate in the recognition of retroviral reverse transcripts. However, why multiple sensors might be required and their relative importance in *in vivo* control of retroviral infection are not known. Here, we show that DDX41 primarily senses the DNA/RNA hybrid generated at the first step of reverse transcription, while cGAS recognizes dsDNA generated at the next step. We also show that both DDX41 and cGAS are needed for the antiretroviral innate immune response to murine leukemia virus (MLV) and HIV in primary mouse macrophages and dendritic cells (DCs). Using mice with cell type-specific knockout of the *Ddx41* gene, we show that DDX41 sensing in DCs but not macrophages was critical for controlling *in vivo* MLV infection. This suggests that DCs are essential *in vivo* targets for infection, as well as for initiating the antiviral response. Our work demonstrates that the innate immune response to retrovirus infection depends on multiple host nucleic acid sensors that recognize different reverse transcription intermediates.

## IMPORTANCE

Viruses are detected by many different host sensors of nucleic acid, which in turn trigger innate immune responses, such as type I interferon (IFN) production, required to control infection. We show here that at least two sensors are needed to initiate a highly effective innate immune response to retroviruses—DDX41, which preferentially senses the RNA/DNA hybrid generated at the first step of retrovirus replication, and cGAS, which recognizes double-stranded DNA generated at the second step. Importantly, we demonstrate using mice lacking DDX41 or cGAS that both sensors are needed for the full antiviral response needed to control *in vivo* MLV infection. These findings underscore the need for multiple host factors to counteract retroviral infection.

## INTRODUCTION

Retroviruses are major causes of disease in animals and humans. The initial immune response to retroviruses is critical to the ability of organisms to clear infection, because once viral DNA integrates into the host chromosomes, persistent infections arise, leading to immunodeficiencies, cancers, and other pathologies. The genomes of mammals and other species include many genes that restrict infectious retroviruses. Among the host antiretroviral factors, APOBEC3 proteins play a major role in restricting retrovirus infection, by cytidine deamination of retroviral DNA and by blocking early reverse transcription (1–7).

The retrovirus RNA genome is converted by the viral reverse transcriptase (RT) enzyme first to RNA/DNA hybrids using a tRNA to prime DNA synthesis and then to double-stranded DNA (dsDNA). Reverse transcription thus creates potential ligands for host sensors that recognize foreign nucleic acids. Cellular recognition of these retroviral reverse transcripts activates the innate immune response. For example, depletion of the host cytosolic DNA exonuclease three prime repair exonuclease 1 (TREX1), a DNA exonuclease, increases the type I interferon (IFN) response to HIV and murine leukemia virus (MLV) infection (4, 8, 9). The TREX1-sensitive retroviral reverse transcripts are recognized by cellular DNA sensors such as cyclic GMP-AMP synthase (cGAS), DEAD-box helicase 41 (DDX41), and ALR family members such as IFN-induced 16 (IFI16) in humans and IFI203 in mice (9–13, 61).

cGAS produces the second messenger cyclic GMP-AMP (cGAMP) upon DNA binding, which binds and activates stimulator of IFN genes (STING) (14–16). STING then translocates from the endoplasmic reticulum to a perinuclear compartment and activates TANK-binding kinase 1 (TBK1), which phosphorylates the transcription factor IFN regulatory factor 3 (IRF3), which in turn enters the nucleus, where it induces type I IFN transcription (17–19). DNA binding to DDX41 and the ALRs also induces type I IFN production via the STING pathway (20). It is not understood how DDX41, which belongs to a family of DEAD-box helicase-containing genes commonly thought to bind RNA, participates in the recognition of nucleic acid. Familial and sporadic mutations in human DDX41 lead to acute myeloblastic leukemia and myelodysplastic syndromes (AML/MDS), suggesting that it also functions as a tumor suppressor (21, 22).

While many studies have shown that the loss of any one of these factors decreases the STING-mediated IFN response to cytosolic DNA, it is not known why there are multiple sensors that converge on the same pathway, particularly *in vivo*. Here, we show that DDX41 recognizes the RNA/DNA intermediate generated by reverse transcription and that DDX41 and cGAS act additively to increase the IFN response and limit retroviral infection *in vivo*. Moreover, using mice with cell-type-specific knockout (KO) of DDX41, we show that dendritic cells (DCs) and not myeloid-derived cells are likely the major sentinel cell targets of *in vivo* infection. These studies reveal why multiple nucleic acid sensors are needed to control retroviral infection and underscore the importance of studying their role in *in vivo* infection.

## RESULTS

### DDX41, IFI203, and cGAS play independent but additive roles in the response to MLV infection.

We showed previously that MLV infection caused a rapid increase in IFN-β RNA levels in murine macrophages that is sensitive to the RT inhibitor zidovudine and that TREX1 depletion further increased this response (4, 9). We also showed that depletion of DDX41, IFI203, or cGAS diminished the IFN-β response with and without TREX1; that all three molecules bound MLV reverse-transcribed DNA; and that IFI203 and DDX41 bound to each other and STING but not to cGAS (9). These data suggested that IFI203 and DDX41 work together in a complex to sense reverse transcripts.

**FIG 1:**
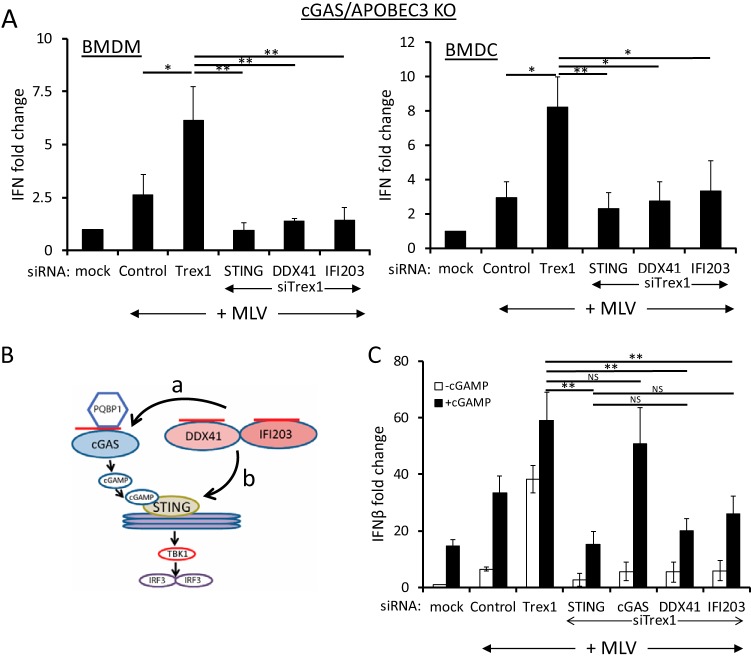
DDX41 and IFI203 work together with cGAS for the maximal antiviral response. (A) Knockdown of STING, DDX41, and IFI203 in *cGas*/*Apobec3* double-knockout BMDMs and BMDCs. Cells were transfected with the indicated siRNAs, and 48 h later, cells were infected with MLV. At 2 hpi, the cells were harvested and examined for IFN-β RNA levels. Knockdown verification of the genes is shown in Fig. S1A in the supplemental material. Values are shown as means ± standard deviations (SDs) from three experiments, each with macrophages and DCs from a different mouse. *P* values were determined by unpaired *t* tests (NS, not significant; *, *P* ≤ 0.05; **, *P* ≤ 0.01). (B) Diagram shows the cGAS-cGAMP-STING pathway. The arrows labeled a and b represent the possible points of DDX41 action; cGAMP addition would rescue DDX41 knockdown if it acted at point a but not if DDX41 acted at point b in the pathway. The red lines represent viral reverse transcripts. (C) cGAMP rescues cGAS but not STING, DDX41, or IFI203 knockdown. NR9456 macrophages were transfected with the indicated siRNAs and 24 h later transfected with cGAMP. At 18 h post-cGAMP treatment, the cells were infected with MLV; IFN-β RNA levels were measured at 2 hpi. Values are shown as means ± SDs from three experiments. Knockdown verification of the genes is shown in Fig. S1B. Mock indicates mock-infected cells.

We hypothesized that DDX41/IFI203 and cGAS play additive but nonredundant roles in the STING/IFN-β activation pathway. To determine if DDX41/IFI203 and cGAS acted synergistically to generate an anti-MLV response, we tested the effects of DDX41, IFI203, and STING depletion in bone marrow-derived macrophages (BMDMs) and DCs (BMDCs) isolated from *cGas* knockout (KO) mice that also lacked APOBEC3; APOBEC3 depletion leads to increased reverse transcript levels and higher levels of IFN induction and thus greater assay sensitivity (4, 9). After small interfering RNA (siRNA)-mediated knockdown, the cells were infected with MLV and the IFN response was determined at 2 h postinfection (hpi), the time of maximum response (4, 9). Despite the lack of cGAS in these cells, MLV infection induced higher levels of IFN-β RNA that were further increased by TREX1 depletion (compare mock, control, and Trex1 siRNA in Fig. 1A), suggesting that additional sensors of retroviral nucleic acid exist in sentinel cells. DDX41 or IFI203 depletion in *cGas* KO BMDMs and BMDCs diminished the type I IFN response to the same level as STING depletion (Fig. 1A). These data show that the full STING-dependent type I IFN response to MLV reverse transcripts requires both DDX41/IFI203 and cGAS.

10.1128/mBio.00923-18.1 FIG S1  Knockdown verification. Related to Fig. 1. (A) Knockdown of genes in Fig. 1A. The knockdowns of the three target genes (*Ddx41*, *Ifi203*, and *Sting*) were all done in the presence of *Trex1* knockdown, as indicated in the Fig. 1 legend. (B) Knockdown of genes in Fig. 1C. The Fig. 1 legend indicates the details. Download FIG S1, PDF file, 0.5 MB. Copyright © 2018 Stavrou et al. 2018 Stavrou et al. This content is distributed under the terms of the Creative Commons Attribution 4.0 International license.

The factor PQBP1 binds retroviral DNA upon infection and functions upstream of cGAS, since cGAMP addition to PQBP1- or cGAS-depleted cells restores the type I IFN response (23) (diagram in Fig. 1B). To determine if DDX41 worked upstream of cGAS, we tested whether cGAMP also would rescue the IFN response in DDX41-, IFI203-depleted cells. siRNA-mediated depletion of IFI203 and DDX41 plus TREX1 was carried out in NR9456 mouse macrophage cells; cGAS and STING depletion served as positive and negative controls, respectively. At 24 h after siRNA transfection, the cells were left untreated or transfected with cGAMP for 18 h and then infected with MLV for 2 h. cGAMP addition did not restore the IFN-β response in DDX41-, IFI203-, or STING-depleted cells (Fig. 1C). As expected, addition of cGAMP restored the IFN-β response in cGAS-depleted cells (Fig. 1C). Taken together with our previously published results, these data suggested that DDX41/IFI203 functions independently of cGAS to activate the STING pathway (pathway b in Fig. 1B) and that the induction of IFN by the two sensors is additive.

### DDX41 is a cytosolic sensor that acts upstream of IRF3 and TBK1.

DDX41 is found in both the nucleus and the cytoplasm (9). The ALR IFI16 senses herpes simplex virus DNA in the nucleus and then migrates to the cytoplasm, where it signals through STING (24, 25). The ultimate product of reverse transcription is a dsDNA that is transported into the nucleus and integrates into the chromosomes. Unintegrated retroviral DNA persists in the nucleus as 1- or 2-long-terminal-repeat (LTR) circles. To determine if DDX41 sensed nuclear retroviral dsDNA, we treated cells with the integrase inhibitor raltegravir, which increases nuclear unintegrated viral dsDNA levels, and examined the IFN response after MLV infection. Although raltegravir treatment dramatically increased the levels of unintegrated nuclear viral DNA, evidenced by abundant 2-LTR circle formation, this treatment had no effect on IFN-β induction (see Fig. S2A in the supplemental material), supporting DDX41 sensing of retroviral reverse transcription products predominantly in the cytoplasm.

**FIG 2:**
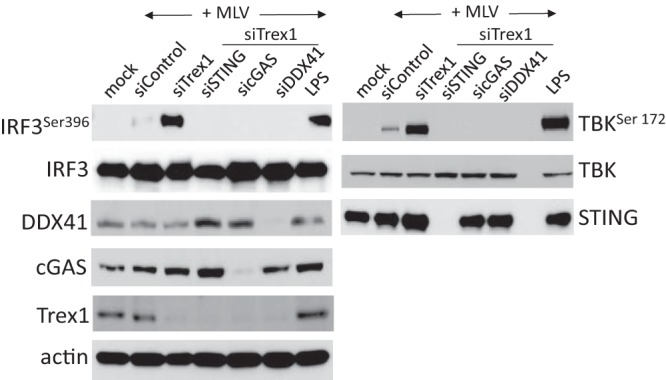
DDX41 acts upstream of IRF3 and TBK1. IRF3 (left) and TBK (right) phosphorylation induced by MLV infection requires DDX41, cGAS, and STING. NR9456 cells were transfected with the indicated siRNAs as well as Trex1 siRNA and 48 h later infected with MLV for 2 h. Control cells were infected but received only control siRNA. The LPS treatments were for 6 h. Equal amounts of protein from the cells were analyzed using the indicated antibodies. Mock indicates mock-infected cells. The TBK1 and IRF3 experiments were performed twice. Shown are representative Western blots.

10.1128/mBio.00923-18.2 FIG S2  DDX41 senses DNA in the cytoplasm via its DEAD domain. Related to Fig. 2. (A) Increasing nuclear dsDNA by inhibiting proviral DNA integration has no effect on DDX41-mediated sensing. NR9456 cells were pretreated with raltegravir (200 nM) and then infected with MLV (MOI of 2) in the presence of drug. DNA and RNA were isolated from infected cells 2 hpi and analyzed for unintegrated viral DNA (2-LTR) or IFN-β RNA levels. Values are shown as means ± SDs from three experiments. *P* values were determined by an unpaired *t* test. *, *P* ≤ 0.05; **, *P* ≤ 0.01; ***, *P* ≤ 0.001. The inset shows levels of *Trex1* RNA knockdown. Download FIG S2, PDF file, 0.2 MB. Copyright © 2018 Stavrou et al. 2018 Stavrou et al. This content is distributed under the terms of the Creative Commons Attribution 4.0 International license.

To test whether DDX41 functioned downstream of STING, TBK1, or IRF3 (Fig. 1B), we siRNA depleted DDX41, cGAS, or STING in NR9456 macrophages, infected them with MLV, and examined IRF3 (Ser396) and TBK1 (Ser172) phosphorylation at 2 h postinfection (hpi); lipopolysaccharide (LPS) treatment served as a positive control. While phospho-TBK1 and -IRF3 were induced in LPS-treated BMDMs and in TREX1-depleted BMDMs in response to MLV, siRNA depletion of DDX41, cGAS, or STING ablated virus-induced TBK1 and IRF3 phosphorylation (Fig. 2). Taken together, these data suggest that DDX41 works in the cytoplasm upstream of STING to induce IFN.

### DDX41 recognizes RNA/DNA hybrid reverse transcription intermediates.

**FIG 3:**
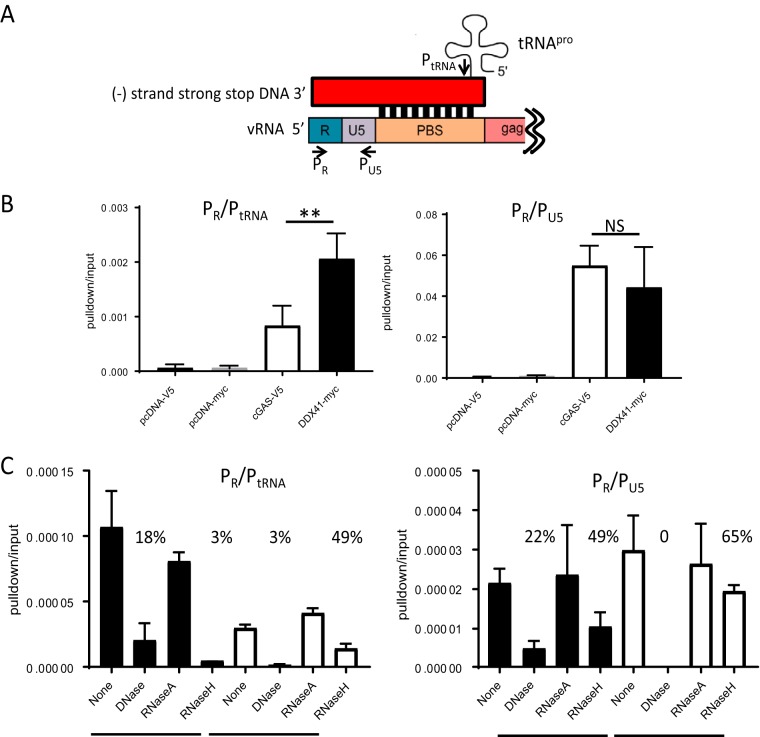
DDX41 preferentially binds RNA/DNA hybrids. (A) Diagram of the tRNA/LTR (P_R_ and P_tRNA_) and strong-stop (P_R_ and P_U5_) primers used to amplify the bound nucleic acid in panel B. The red box represents the newly synthesized viral DNA; shown below is the viral RNA. Abbreviations: vRNA, viral RNA; PBS, primer binding site. (B) DNA pulldown assays with extracts from 293T MCAT1 cells transfected with the indicated constructs and infected with MLV. Shown are the means from 4 independent experiments ± SD. **, *P* ≤ 0.01 (unpaired *t* test); NS, not significant. (C) DNA pulldown assays were conducted as for panel B, except that prior to the reverse transcription/RT-qPCR, the nucleic acids were treated with the indicated nucleases. Shown are the averages from two experiments done in triplicate. The numbers above the columns show percent nucleic acid pulldown relative to no nuclease.

Retroviruses generate several replication intermediates which could be sensed as foreign—tRNA-bound DNA/RNA hybrids, single-stranded DNA (ssDNA), and dsDNA. We used three approaches to examine which reverse transcription products were sensed by DDX41. First, to determine if DDX41 or cGAS bound to tRNA primer-containing reverse transcription intermediates, 293T cells stably expressing the MLV receptor MCAT1 were transiently transfected with DDX41 or cGAS expression constructs and infected with MLV and pulldown experiments were performed. After the pulldown experiments, DNA was isolated from half of each sample and subjected to PCR amplification with primers that detect early reverse transcripts (strong-stop primers P_R_ and P_U5_), while cDNA was prepared from the remaining half and amplified with P_R_ and a 3′ primer specific to tRNA^Pro^ (P_tRNA_), the tRNA used by MLV RT to prime reverse transcription (Fig. 3A). DDX41 bound to >2-fold more tRNA^Pro^-containing reverse transcripts, while DDX41 and cGAS equally precipitated a product that amplified strong-stop DNA (Fig. 3B).

**FIG 4:**
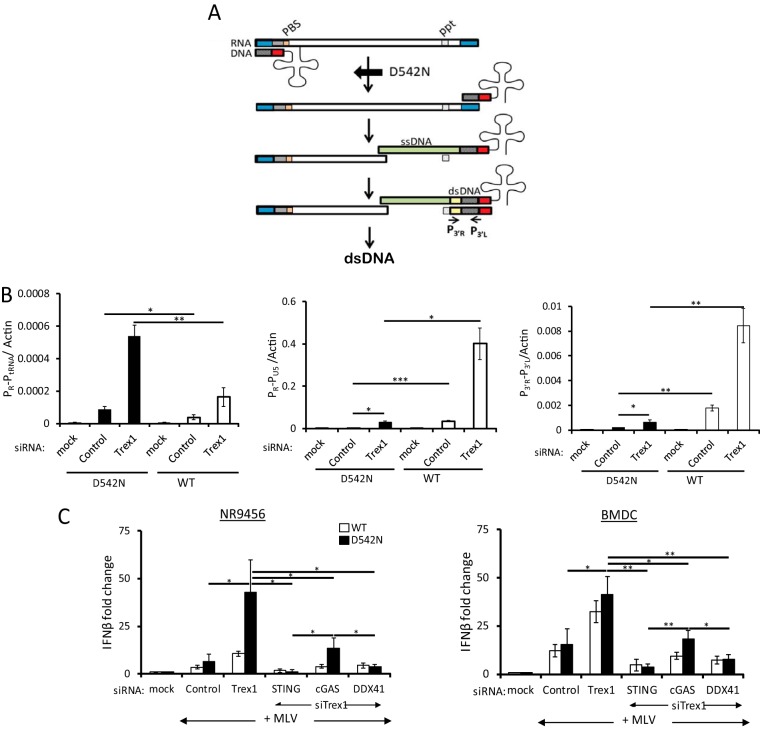
MLV^D542N^ reverse transcripts are sensed by DDX41. (A) Diagram of the early stages of reverse transcription. The RNase H^D542N^ mutation allows initial reverse transcription, but because of the loss of RNase H activity, strong-stop DNA cannot translocate to the 3′ end of the viral RNA to initiate transcription of the full-length viral dsDNA; the tRNA primer is also not degraded. P_3′R_ and P_3′L_ denote the 3′ LTR primers used in panel B. Abbreviations: PBS, primer binding site; ppt, polypurine tract; ssDNA, single-stranded DNA; ds, double-stranded DNA. (B) NR9456 cells treated with the indicated siRNAs were infected with D542N or wild-type virus, and 2 hpi, RNA was subjected to RT-qPCR with primers to the tRNA-containing RNA/DNA hybrid (P_R_ and P_tRNA_, Fig. 3A), while DNA was subjected to qPCR with strong-stop DNA (P_R_ and P_U5_, Fig. 3A) or late reverse transcripts (P_3′R_ and P_3′L_ primers [A]). Shown are the means ± SDs from 3 independent experiments. *, *P* ≤ 0.05; **, *P* ≤ 0.01; ***, *P* ≤ 0.001 (unpaired *t* test). (C) Recognition of RNA-DNA hybrids requires DDX41. NR9456 cells (left) or BMDCs (right) were transfected with the indicated siRNAs and infected with wild-type MLV or MLV^D542N^ for 2 h, and the levels of IFN-β RNA were measured. Shown are the means ± SDs from 3 independent experiments. *, *P* ≤ 0.05; **, *P* ≤ 0.01; ***, *P* ≤ 0.001 (unpaired *t* test). Knockdown of the genes is shown in Fig. S3. Mock indicates mock-infected cells.

Second, we treated the DDX41- and cGAS-bound nucleic acids with RNase H, which degrades RNA in DNA/RNA hybrids as well as the tRNA primer; DNase I, which cleaves dsDNA 100- and 500-fold better than RNA/DNA hybrids and ssDNA, respectively; and RNase A, which degrades ssRNA under high-salt conditions. DDX41 again more efficiently precipitated the RNA/DNA hybrid, and RNase H treatment reduced the amount of DDX41-precipitated nucleic acid to 3%. In contrast, RNase H digestion only modestly affected cGAS pulldown of the product amplified with the P_R_/P_tRNA_ primer pair, suggesting that DDX41 preferentially bound the RNA/DNA hybrid while cGAS bound to tRNA primer-bound dsDNA generated after strand translocation (Fig. 3C, left panel; diagram in Fig. 4A). In support of this, DNase I treatment abolished cGAS-mediated pulldown of both the P_R_/P_tRNA_- and P_R_/P_U5_-amplifiable products, while DDX41-mediated precipitation of nucleic acid (Fig. 3C, left and right panels) was affected to a lesser extent. RNase A digestion in high salt had no effect on any of the pulldowns.

10.1128/mBio.00923-18.3 FIG S3  Knockdown verification. Related to Fig. 4. Knockdown of genes in Fig. 4C. The Fig. 4 legend indicates the details. Download FIG S3, PDF file, 0.2 MB. Copyright © 2018 Stavrou et al. 2018 Stavrou et al. This content is distributed under the terms of the Creative Commons Attribution 4.0 International license.

Finally, we used a viral mutant lacking RNase H activity. During reverse transcription, RT’s RNase H moiety degrades the positive-strand RNA genome after the synthesis of minus-strand [(−)-strand] DNA (Fig. 4A) (26). RNase H mutations attenuate the RNase H function without diminishing the polymerase activity. RNase H^D542N^ synthesizes tRNA^Pro^-primed (−)-strand strong-stop DNA while retaining ~10% of the wild-type (WT) levels of RNase H enzymatic activity. As a result, the RNA remains “frozen” in a DNA/RNA hybrid and (−)-strand strong-stop DNA does not efficiently translocate to the 3′ end of the viral RNA to initiate full-length (−)-strand DNA synthesis (Fig. 4A) (27, 28). We engineered the D542N mutation into an MLV molecular clone (MLV^D542N^) and used this virus to infect NR9456 cells. MLV^D542N^ generated almost 3-fold more reverse transcription products retaining the tRNA primer than did the wild-type virus, reflecting its poorer ability to translocate the negative-strand strong-stop DNA to the 5′ end of the RNA and degrade the tRNA primer and its known increased DNA polymerase activity relative to wild-type virus (P_R_-P_tRNA_, Fig. 4B) (27, 29). The mutation dramatically attenuated reverse transcription detected with the strong-stop (P_R_-P_U5_) primers compared to wild-type virus, since these primers detect R-U5 DNA present in (−)- and (+)-strand strong-stop as well as full-length (−)-strand DNA; late reverse transcription (P_3′R_-P_3′L_) products were also reduced compared to wild-type virus (Fig. 4B). Interestingly, Trex1 depletion led to increases in reverse transcription products retaining tRNA from both the wild-type and MLV^D542N^ viruses, suggesting that negative-strand strong-stop DNA is also degraded by this cellular exonuclease (Fig. 4B); it has been previously shown that TREX1 degrades ssDNA and dsDNA and DNA in RNA/DNA hybrids (8, 30, 31).

To determine whether DDX41 or cGAS was better able to recognize the early DNA/RNA reverse transcription product, we infected NR9456 cells or primary BMDCs with MLV^D542N^ after treatment with *Trex1* siRNA alone or in combination with *Sting*, *Ddx41*, or *cGas* siRNAs. MLV^D542N^ caused about a 5- and 2-fold increase in the IFN response compared to wild-type virus in the *Trex1* siRNA-treated and untreated NR9456 cells, respectively (Fig. 4C, left panel). DDX41 knockdown diminished the response to both viruses to the same levels as seen with STING knockdown in both NR9456 cells and primary BMDCs (Fig. 4C). Depletion of cGAS reduced but did not completely abrogate the TREX1-dependent response to MLV^D542N^ in NR9456 or primary BMDCs (Fig. 4C). The response to the RNase H mutant virus in cGAS-deficient cells was likely due to DDX41-mediated recognition of the RNA/DNA hybrid in these cells (Fig. 4C).

The results from these 3 complementary approaches indicate that DDX41 preferentially senses the RNA/DNA hybrid generated during the earliest stage of reverse transcription while cGAS preferentially recognizes dsDNA generated at the next step.

### DDX41 is required for the IFN response in both macrophages and DCs.

**FIG 5:**
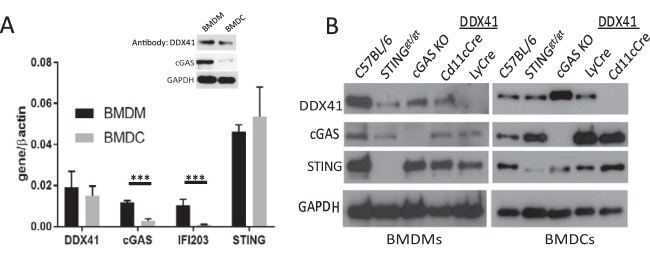
Characterization of *Ddx41* knockout BMMs and BMDCs. (A) Basal expression of the different sensors in wild-type BMDCs and BMDMs. Shown are the averages and SDs for cells isolated from 3 different mice. The inset shows Western blot analysis of 40 µg each of extracts from BMDMs and BMDCs, probed with antisera against DDX41, cGAS, and GAPDH. (B) Relative expression of DDX41 in BMDMs and BMDCs. Forty micrograms of protein from cells isolated from mice of the indicated genotypes was analyzed by Western blotting with antisera to the indicated proteins. Gels are representative of 3 independent experiments. Averages from the 3 experiments are shown in in Fig. S4B.

Macrophages and DCs have both been implicated in the antiretroviral innate immune response. We examined DDX41 expression in BMDMs and BMDCs and found that it was expressed in both cell types at both the RNA and the protein level (Fig. 5A). Interestingly, in contrast to DDX41 expression, *cGas* and *Ifi203* RNA levels and cGAS protein levels were significantly higher in wild-type BMDMs than in BMDCs, suggesting that the sensors used to detect nucleic acid might be cell type specific (Fig. 5A). The lack of anti-IFI203-specific antisera prevented us from determining whether its protein levels in BMDMs and BMDCs reflected the RNA levels.

**FIG 6:**
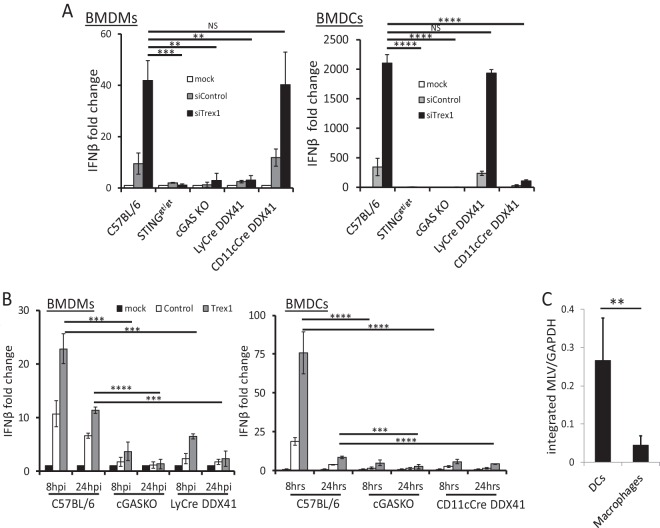
MLV and HIV induce a DDX41-dependent IFN-β response in BMDMs and BMDCs. (A) BMDMs and BMDCs isolated from mice of the indicated genotypes were infected with MLV, and at 2 hpi, IFN-β levels were measured. The data in the graph are the averages from 3 different experiments, each with macrophages and DCs from a different mouse. NS, not significant; *, *P* ≤ 0.05; **, *P* ≤ 0.01; ***, *P* ≤ 0.001 (unpaired *t* test). Verification of the knockdown is shown in Fig. S5B. ELISAs measuring IFN-β protein are shown in Fig. S5A. (B) HIV pseudotype infection in *cGas* and *Ddx41* KO BMDMs and relative infection of BMDMs and BMDCs by MLV. HIV cores pseudotyped with ecotropic MLV glycoproteins were used to infect BMDMs (left) or BMDCs (right) from mice of the indicated genotypes. Trex1 knockdowns are shown in the right panels. Shown are the averages from 3 independent experiments. ***, *P* ≤ 0.001; ****, *P* ≤ 0.0001 (unpaired *t* test). (C) DCs and macrophages were isolated from MLV-infected mice at 16 dpi, and levels of integrated MLV were determined by qPCR. **, *P* ≤ 0.01. Mock indicates mock-infected cells.

10.1128/mBio.00923-18.4 FIG S4  Characterization of *Ddx41* KO mice. Related to Fig. 5 and 6. (A) Map of the DDX41 locus and inserted loxP sites. Expression of Cre recombinase results in the deletion of exons 7 to 9. (B) Quantification of DDX41, cGAS, and STING protein in various knockout mouse cells. Shown are the means ± SDs for 3 independent Western blotting assays of cells from 3 different mice of each strain. *, *P* ≤ 0.05 compared to BL/6, *Sting^gt/gt^*, and CD11cCreDDX41 (unpaired *t* test). (C) Basal IFN-β RNA levels in *Ddx41* KO BMDMs and BMDCs. RNA was isolated from BMDMS of 3 mice and BMDCs of 2 mice each of the indicated genotypes, and qPCR was performed for IFN-β levels, using a standard curve to measure relative levels. Shown are the means ± SDs. Abbreviations: ND, not done. (D) PBMCs from 4 mice of each genotype were stained with conjugated anti-CD11c (DC) or anti-F4/80 (macrophage) antibodies and analyzed by FACS. (E) Treatment of DDX41 KO BMDMs and BMDCs with different ligands. BMDMs and BMDCs from the *cGas*, LyCre DDX41, and CD11cCre-DDX41 KO and *Sting^gt/gt^* mice were treated with the indicated ligands, as described in Materials and Methods. RNA was isolated after 6 h of treatment for all ligands and subjected to RT-qPCR. Shown are the averages from 2 experiments (triplicate technical replicates) with cells isolated from different mice. Download FIG S4, PDF file, 0.3 MB. Copyright © 2018 Stavrou et al. 2018 Stavrou et al. This content is distributed under the terms of the Creative Commons Attribution 4.0 International license.

To determine whether DDX41 was important for IFN induction in these cell types, we used mice with a knocked-in floxed *Ddx41* allele, in which the loxP sites flank exons 7 and 9 (Fig. S4A). We crossed these mice with CMV-Cre mice, but no complete KO pups were generated, suggesting that germline loss of *Ddx41* causes embryonic lethality. We then crossed these mice with CD11cCre and LyCre transgenic mice to generate DC- and myeloid lineage-specific KOs, respectively. We also used BMDMs and BMDCs from *cGas* KO mice and *Sting^gt/gt^* mice, which encode a mutant STING protein incapable of signaling and whose protein levels are greatly reduced (32). BMDMs from the LyCre-DDX41 mice and BMDCs from the CD11cCre-DDX41 mice were deficient in DDX41 RNA and protein but had wild-type levels of STING and cGAS (Fig. 5B and S4B). Additionally, DDX41 protein levels were significantly higher in the *cGas* KO BMDCs but not BMDMs (Fig. 5B and S4B). Basal levels of IFN were not also not affected by loss of DDX41 (Fig. S4C), and fluorescence-activated cell sorting (FACS) analysis demonstrated that DDX41 deficiency did not affect overall percentages of peripheral blood DCs or macrophages in the CD11cCre-DDX41 or LyCre-DDX41 mice (Fig. S4D). To ensure that DDX41 loss did not affect all innate immune responses, BMDCs and BMDMs from CD11cCre-DDX41 and LyCre-DDX41 mice were treated with the Toll-like receptor 4 (TLR4) ligand lipopolysaccharide (LPS), the TLR3/MAVS pathway ligand poly(I⋅C), and cGAMP; cells from *cGas* KO, *Sting^gt/gt^*, and C57BL/6N mice served as controls. The responses to LPS and poly(I⋅C) were similar to those of the wild type in CD11cCre-DDX41 BMDCs, LyCre-DDX41 BMDMs, and *cGas* KO and *Sting^gt/gt^* BMDMs and BMDCs (Fig. S4E). cGAMP responses were reduced only in *Sting^gt/gt^* cells, as previously reported (33).

We then used these cells to examine the response to MLV infection. Mouse BMDCs or BMDMs lacking DDX41 showed little or no increase in type I IFN RNA (Fig. 6A) or protein (Fig. S5A) in response to MLV, even when TREX1 levels were reduced by siRNA treatment. BMDCs and BMDMs from *Sting^gt/gt^* and *cGas* KO mice also had an abrogated antiviral IFN-β response under the same conditions (Fig. 6A and S5A). We also tested whether the response to HIV-1 was defective in the various mouse knockout cells, using pseudoviruses bearing the ecotropic MLV envelope; the IFN-β RNA response to HIV was diminished in both *Ddx41* and *cGas* KO BMDMs and BMDCs (Fig. 6B). Thus, both sensors are required for the full type I IFN response to both MLV and HIV in mouse cells.

10.1128/mBio.00923-18.5 FIG S5  Loss of DDX41 decreases the IFN response to MLV and HIV infection. Related to Fig. 6. (A) BMDMs and BMDCs from (C57) cGAS, LyCre-DDX41, and CD11cCre-DDX41 mice were treated with siCont or siTrex and then infected with MLV^glycoGag^. Four hours later, supernatants were used to perform an ELISA for IFN-β levels. Shown are the averages from 3 independent experiments. ****, *P* ≤ 0.0001 (unpaired *t* test). (B) Verification of Trex1 knockdown in BMDCs and BMDMs in Fig. 6A. (C) Verification of the knockdowns in BMDMs and BMDCs in Fig. 6B. Download FIG S5, PDF file, 0.1 MB. Copyright © 2018 Stavrou et al. 2018 Stavrou et al. This content is distributed under the terms of the Creative Commons Attribution 4.0 International license.

The TREX1-/DDX41-dependent IFN-β response to MLV infection was much higher in BMDCs than in BMDMs; there was a 2,000-fold increase in IFN-β RNA in BMDCs compared to BMDMs, where the response was about 40-fold (compare *y* axes in Fig. 6A and B). To determine if this was due to increased infection, we isolated splenic DCs and macrophages from MLV-infected C57BL/6 mice at 16 days postinoculation (dpi), as well as *ex vivo* infected BMDCs and BMDMs from mice of all the genotypes. Integrated MLV DNA was analyzed by quantitative PCR (qPCR) with a B1 repeat- and MLV LTR-specific primers. MLV infection of DCs was about 1 order of magnitude higher than that of macrophages, after either *in vivo* or *ex vivo* infection, independent of the mouse genotype (Fig. 6C and S6, respectively). Thus, while macrophages can be infected, sustain reverse transcription, and mount a response to viral nucleic acids, DCs are more infected and respond more robustly to infection.

10.1128/mBio.00923-18.6 FIG S6  BMDCs are more infected by MLV than BMMs. Related to Fig. 6. BMDMs and BMDCs from mice of the indicated genotypes were infected with MLV, and at 24 and 48 hpi, DNA was isolated from cells and subjected to qPCR, using one primer to mouse genomic DNA and the other to the viral long terminal repeat. Values are shown, as well as means ± standard errors of the means for 2 experiments done for each cell type. Download FIG S6, PDF file, 0.1 MB. Copyright © 2018 Stavrou et al. 2018 Stavrou et al. This content is distributed under the terms of the Creative Commons Attribution 4.0 International license.

### Full suppression of MLV infection *in vivo* requires both DDX41 and cGAS.

**FIG 7:**
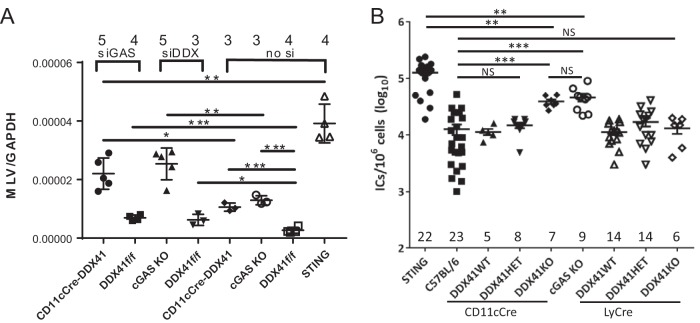
*In vivo* control of retrovirus infection requires both cGAS and DDX41. (A) Mice of the indicated genotype received footpad injections of siRNAs. Forty-eight hours postinjection, the mice were injected with MLV. RNA was harvested from the draining lymph node at 24 hpi and analyzed for MLV RNA by reverse transcription qPCR; values were normalized to GAPDH RNA. Knockdown verification is shown in Fig. S7. ****, *P* ≤ 0.00001; ***, *P* ≤ 0.0001; **, *P* ≤ 0.001; *, *P* ≤ 0.01 (unpaired *t* test). The number of mice in each group is shown above the graph. (B) Newborn mice of the indicated genotypes were inoculated with MLV. At 16 dpi, virus titers in spleen were measured. Each point represents an individual mouse. The numbers of mice analyzed in each group are shown on the graph; each of the groups of mice came from 4 to 10 independent litters. Horizontal bars represent the averages. **, *P* ≤ 0.001; NS, not significant (Mann-Whitney *t* test).

To determine whether DDX41 and cGAS functioned *in vivo* to suppress infection, we subcutaneously inoculated the CD11cCre-DDX41 and *cGas* KO mice with MLV and measured infection levels in the draining lymph node; wild-type (*Ddx41f/f* mice without Cre) and *Sting^gt/gt^* mice served as controls. The CD11cCre-DDX41 and *cGas* KO mice showed significantly higher levels of infection than the wild-type mice, while *Sting^gt/gt^* mice had the highest level of infection (Fig. 7A).

10.1128/mBio.00923-18.7 FIG S7  *In vivo* control of retrovirus infection requires both cGAS and DDX41. Related to Fig. 7. (A) Knockdown verification of the *in vivo* siRNA experiment presented in Fig. 7A. (B) Genotypes of the mice tested for MLV infection in Fig. 7B. Parental mice were Cre^+/−^ DX41^+/−^. Cre refers to either LyCre or CD11cCre, as indicated in the text. Yellow highlighted boxes refer to homozygous, and white boxes refer to heterozygous, *Ddx41* tissue-specific knockouts; gray boxes refer to WT mice. Download FIG S7, PDF file, 0.1 MB. Copyright © 2018 Stavrou et al. 2018 Stavrou et al. This content is distributed under the terms of the Creative Commons Attribution 4.0 International license.

Next, we tested whether DDX41 and cGAS acted synergistically *in vivo*. We treated CD11cCre-DDX41 and wild-type mice with cGAS siRNAs and *cGas* KO and wild-type mice with DDX41 siRNAs; mice injected with the *in vivo* transfection reagent Invivofectamine alone served as controls. At 48 h after siRNA treatment, the mice were infected with MLV in the same footpad, and at 24 hpi, RNA was isolated from the draining lymph node and examined for MLV RNA levels (Fig. 7A) and the extent of gene knockdown (Fig. S7). CD11cCre-DDX41 mice that received the cGAS siRNA and *cGas* KO mice that received the DDX41 siRNA were infected at >8-fold-higher levels than wild-type mice receiving no siRNA and at >3-fold-higher levels than wild-type mice receiving the DDX41 or cGAS siRNA. Infection levels in the CD11cCre-DDX41/cGAS siRNA group were not statistically different from those in the *cGas* KO/DDX41 siRNA group (Fig. 7A). Wild-type mice receiving the DDX41 or cGAS siRNAs were >2-fold more infected than untreated wild-type mice and were not statistically different from each other. *Sting^gt/gt^* mice had the highest levels of infection, about 2-fold higher than *cGas* KO/DDX41 siRNA or CD11cCre-DDX41/cGAS siRNA mice.

We also examined whether DDX41 expression in BMDMs or BMDCs was important to suppress long-term *in vivo* infection. Newborn offspring from crosses between LyCre-DDX41^+/−^ and CD11cCre-DDX41^+/−^ mice as well as newborn C7BL/6, *Sting^gt/gt^*, and *cGas* KO pups were inoculated with MLV, and at 16 dpi, virus titers in their spleens were measured; this time point has been used extensively by us and others to examine MLV infection (4, 5, 7, 9, 34, 35). The genotyping of the intercrossed mice was carried out subsequent to measuring the virus titers. We thus compared infection levels between mice with a total lack of DDX41 due to full knockout of the gene in the specific compartment and mice with only one knockout allele and mice with no knockout of *Ddx41* (Fig. S6B).

Mice with complete knockout of *Ddx41* in DCs showed 5-fold-higher infection than either wild-type mice or mice heterozygous for the DDX41 knockout allele in this cell type (Fig. 6). *cGas* KO mice were also more infected, also to about 5-fold-higher levels than wild-type mice, and the level of infection was the same as that of the CD11cCre-DDX41 mice (Fig. 6). In contrast, *Sting^gt/gt^* mice were most highly infected with MLV, about 10-fold higher than wild-type mice (Fig. 6). This confirms that cGAS and DDX41 are both required for full sensing of retroviral reverse transcripts and for the control of virus *in vivo*. Surprisingly, MLV infection of mice with complete knockout of *Ddx41* in macrophages was the same as that in wild-type mice or heterozygotes (Fig. 6). Thus, although DDX41 sensed MLV infection in macrophages *in vitro* and *ex vivo*, this response *in vivo* was not sufficient to control infection.

## DISCUSSION

The host factor APOBEC3, which both blocks reverse transcription and causes lethal mutation of the viral genome, is likely the first line of defense against retroviruses, although incoming retroviruses do generate ligands that activate the innate immune system (9). We recently proposed that the major role for cytosolic sensing of reverse transcripts that escape the APOBEC3-mediated reverse transcription block is to induce expression of IFN-stimulated genes (ISGs), including APOBEC3 itself (9). The most highly studied of these sensors, cGAS, is clearly a critical component of the foreign DNA recognition pathway, leading to STING activation and the type I IFN response. However, the role of other sensors implicated in the response to DNA generated during pathogen infection remains controversial. These include DDX41 as well as members of the ALR family (4, 10, 13, 36, 37). Here, we show that DDX41 is a critical sensor of viral nucleic acids generated during reverse transcription and is required to control *in vivo* infection.

Retroviruses are unique in generating multiple different forms of nucleic acid during their replication in the cytoplasm which can be recognized as “foreign” by the host cell. DDX41 is likely recognizing the DNA/RNA hybrid generated in the first step of retrovirus replication, and cGAS is likely recognizing the dsDNA generated after strand translocation. While we showed that DDX41 and cGAS KO BMDMs or BMDCs showed diminished responses to transfected synthetic dsDNA or DNA/RNA molecules, DDX41 preferentially precipitated RNase H-sensitive, DNA/RNA hybrid reverse transcripts generated during MLV infection, and only depletion of DDX41 specifically reduced the IFN response to the RNase H mutant virus, which generates more RNA/DNA hybrids than does wild-type MLV. In contrast, cGAS precipitated DNase I-sensitive reverse transcripts, and cGAS depletion did not completely abrogate the type I IFN response to the RNase H mutant generated at the first step of reverse transcription. Whether the presence of the tRNA primer bound to DNA/RNA hybrids or dsDNA plays a role in the recognition by DDX41 or cGAS, respectively, is currently not known.

*DDX41* belongs to a family of RNA helicases, with distinct DEAD/H-box (Asp-Glu-Ala-Asp/His) domains, whose members have been implicated in translation, ribosome biogenesis, nuclear-cytoplasmic transport, organelle gene expression, and pre-mRNA splicing (38–40). *DDX41* was recently identified as a tumor suppressor gene in familial and sporadic myelodysplastic syndrome/acute myeloid leukemia (MDS/AML), as well as other hematological malignancies (21, 22, 39). In MDS/AML, DDX41 is thought to interact with spliceosomal components and alter splicing, resulting in the inactivation of tumor suppressor genes or alterations in the balance of gene isoforms, although whether this occurs through protein-RNA, protein-DNA, or protein-protein interactions is not known. Our data showing that DDX41 interacts with RNA/DNA hybrids are consistent with the known ability of DEAD-box proteins’ recognition of RNA and suggest that DDX41 may have evolved an antiviral cytoplasmic activity that takes advantage of its unique ability to interact with both RNA and DNA, as well as proteins. Another DEAD-box helicase, DDX3, was also recently implicated in the sensing of HIV RNA in DCs (41). However, DDX3 sensed abortive RNA transcribed from integrated proviruses, whereas DDX41 sensing occurred in the presence of the integrase inhibitor raltegravir, confirming that it works at a very early step of infection.

A previous study suggested that DDX41 might be the initial cytosolic sensor in BMDMs and that type I IFNs induced by DDX41 sensing lead to increased expression of cGAS, which is an ISG (42). However, at 2 hpi, cGAS- and DDX41-deficient cells showed similar decreased IFN-β RNA levels after MLV infection, suggesting that both sensors are needed for the initial response. We showed previously that DCs get infected by MLV (5), and here, we demonstrate that DDX41 in DCs but not macrophages was required for *in vivo* control of virus infection. The innate immune response initiated by DDX41 sensing of MLV in DCs may be due to higher levels of infection than in macrophages or because DCs are more effective at initiating antiviral responses. Whether the cGAS-dependent response is also required to control *in vivo* infection primarily in DCs is not known. Nevertheless, the results presented here contradict studies suggesting that DCs do not get infected but serve only as carriers that deliver intact retroviral virions to lymphocytes (43–45).

Previous work suggested that only cGAS is important for sensing retroviruses via the STING pathway (12, 46). These studies used vesicular stomatitis virus (VSV) G protein-pseudotyped HIV or MLV cores. Both HIV and MLV naturally enter cells from a neutral compartment, and it is possible that the use of VSV G, which directs entry to an acidic compartment, might affect the accessibility of different sensors to the reverse transcription complex. Additionally, these studies tested embryonic fibroblasts or BMDMs. However, as we demonstrated previously and our *ex vivo* and *in vivo* studies here demonstrate, DCs are likely the important targets of retroviruses (5, 47). Indeed, we also show here that endogenous cGAS expression in DCs, the relevant cell type for controlling MLV infection *in vivo*, is ~4-fold lower than that seen in macrophages, which could also account for the differences in our results with previous studies. Similar differences in cGAS expression occur in human macrophages and DCs (48).

Finally, earlier studies did not examine the effects of the different sensors on *in vivo* infection. We show here that effective *in vivo* control of MLV infection via the STING pathway requires both DDX41 and cGAS. However, as we and others have shown, the retrovirus capsid likely protects the reverse transcription complex from host sensors and other restriction factors, including APOBEC3 proteins (9, 49, 61). This may explain why mice lacking DDX41 or cGAS show only 5-fold-higher infection than wild-type mice; even STING-deficient mice show only 10-fold-higher infection (Fig. 7) (9). Our data are consistent with a requirement for both DDX41 and cGAS, the former perhaps in complex with IFI203, to achieve the full antiviral IFN response to retroviral reverse transcripts not protected by capsid or blocked by APOBEC3 proteins. Whether DDX41 requires interaction with IFI203 to achieve maximum effect *in vivo* will also be important to determine; however, *Ifi203* shares a high degree of identity in the noncoding as well as coding regions with several other genes in the *Alr* locus, making a gene-specific knockout difficult to achieve (50). How nucleic acid-bound DDX41 activates STING is also not yet understood, although the two molecules are known to directly bind each other (9, 13).

Our data suggest that there are multiple cytosolic sensors that recognize the different types of nucleic acids generated during retrovirus infection. Understanding the initial host response to infection by retroviruses is critical to our ability to determine how these viruses establish persistent infection as well the discovery of novel approaches to intervene in these infections.

## MATERIALS AND METHODS

### Mice.

Mice were bred at the University of Pennsylvania and the University of Illinois at Chicago. DDX41-flp mice (C57BL/6N) were constructed by TaconicArtemis GmbH and were derived by the University of Pennsylvania Transgenic and Chimeric Mouse Facility from *in vitro* fertilization of C57BL/6N embryos with sperm from a single male. LyCre [B6.129P2-Lyz2tm1(cre)Ifo/J] and *Sting^gt/gt^* (C57BL/6J-Tmem173gt/J) mice were purchased from the Jackson Laboratory. CD11cCre mice [B6.Cg-Tg(Itgax-cre)1-1Reiz/J] were provided by Yongwon Choi, and *cGas* KO mice *(Mb21d1^tm1d(EUCOMM)Hmgu^)* were provided by Michael Diamond and Skip Virgin (51). Apobec3 knockout mice were previously described (52). cGas/Apobec3 double-knockout mice were generated by intercrossing the two strains. All mice were housed according to the policies of the Institutional Animal Care and Use Committee (IACUC) of the University of Pennsylvania and of the Animal Care Committee (ACC) of the University of Illinois at Chicago (UIC); all studies were performed in accordance with the recommendations in the *Guide for the Care and Use of Laboratory Animals* of the National Institutes of Health (62). The experiments performed with mice in this study were approved by the University of Pennsylvania IACUC (protocol no. 801594) and UIC ACC (protocol no. 15-222).

### FACS analysis and sorting.

Peripheral blood mononuclear cells were stained with anti-mouse F4/80-fluorescein isothiocyanate (FITC) (BioLegend) and anti-mouse CD11c-phycoerythrin (PE) (BD Bioscience) antibodies. Cells were processed using a Beckman Coulter CyAn ADP flow cytometer. Results were analyzed using FlowJo software.

### Virus.

Moloney MLV (MMLV) and MLV^glycoGag^ mutant viruses were harvested from stably infected NIH 3T3 fibroblasts, as previously described (53). Titers of all virus preparations were determined on NIH 3T3 cells and analyzed by RT-qPCR for viral RNA levels as previously described (4). To generate the RNase H mutant virus, the D524N mutation previously described by Blain and Goff (27) was introduced into the wild-type (WT) MLV infectious clone p63.2 (54) by site-directed mutagenesis using the QuikChange II XL site-directed mutagenesis kit (Agilent Technologies) and the primers 5′-ACCTGGTACACGAATGGAAGCAGTCTCTTAC-3′/5′-GTAAGAGACTGCTTCCATTCGTGTACCAGGT-3′; the mutation was verified by sequencing. The p63.2 and p63.2^D524N^ plasmids were transfected in 293T cells using Lipofectamine 3000 (Invitrogen). The media of the transfected cells were harvested 48 h posttransfection, centrifuged at 500 × *g* for 10 min at 4°C, filtered through a 0.45-µm filter, and treated with DNase I recombinant RNase Free (Roche). Virus levels were determined by the QuickTiter MuLV Core Antigen enzyme-linked immunosorbent assay (ELISA) kit (MuLV p30) (Cell Biolabs, Inc.) and by titer determination on NIH 3T3 cells stably transfected with pRMBNB, which expresses the MLV *gag* and *pol* genes (28).

### BMDM and BMDC cultures.

BMDMs and BMDCs were isolated from hind limbs of 10- to 12-week-old *cGas* KO, *Sting^gt/gt^*, LyCre-DDX41, CD11cCre-DDX41, and C57BL/6 mice as previously described (55). BMMs were cultured in Dulbecco modified Eagle medium (DMEM) supplemented with 10% fetal bovine serum (FBS), 10 ng/ml macrophage colony-stimulating factor (Invitrogen), 1 mM sodium pyruvate, 100 U/ml penicillin, and 100 µg/ml streptomycin; were harvested 7 days after plating; and were seeded in 96-well plates for infection assays. BMDCs were cultured in RPMI supplemented with 5% FBS and differentiated with recombinant murine granulocyte-macrophage colony-stimulating factor (20 ng/ml; Invitrogen). Both procedures result in cultures that are ~80% to 85% pure.

### cGAMP stimulation of macrophages.

Knockdowns with the indicated siRNAs were performed in NR9456 macrophages (immortalized macrophage cell line derived from C57BL/6 wild-type mice) (56) (BEI Resources, NIAID, NIH) using RNAiMAX, as previously described (9). The next day, cells were transfected with Lipofectamine 2000 (Invitrogen) and 4 µg of cGAMP (InvivoGen), and 16 h later, the cells were infected with MLV^glycoGag^ and harvested 2 hpi. RNA isolation and qPCR analysis were performed as previously described (9).

### Virus infection of macrophages and DCs.

NR9456 macrophages, BMDMs, and BMDCs were siRNA transfected. At 48 h after transfection, the cells were infected with wild-type, MLV^glycoGag^, or D542N mutant MMLV (multiplicity of infection [MOI] of 2) and harvested at the indicated times after infection. For some experiments, the cells were treated with 200 nM raltegravir for 2 h prior to infection and then infected with MLV^glycoGag^ virus in the presence of drug. Cells were harvested 2 hpi; RNA isolation and RT-PCR were performed as previously described (9). Primers used for detection of actin, Trex1, and IFN-β were previously described (4, 57). Primers to amplify the MLV 2-LTR closed circles are 5′-GAGTGAGGGGTTGTGGGCTCT-3′/5′-ATCCGACTTGTGGTCTCGCTG-3′ (58). Primers used to amplify late reverse transcripts (P_3′R_/P_3′L_) are 5′-TAACGCCATTTTGCAAGGCA-3′/5′-GAGGGGTTGTGGGCTCTTTT-3′; strong-stop DNA primers were reported previously (4).

### BMDM and BMDC treatment with synthetic ligands.

BMDMs and BMDCs isolated from C57BL/6, Sting^gt/gt^, cGas KO, LyCre-DDX41, and CD11c DDX41 mice were transfected with 2 ng/µl poly(I⋅C) (Sigma) and 4 ng of cGAMP using Lipofectamine 3000; cells were also treated with 1 ng/µl LPS (Sigma). The cells were harvested at 6 h posttreatment. RNA was isolated, and cDNA was generated using the SuperScript III kit (Invitrogen). RT-PCR was performed to measure IFN-β RNA levels, as previously described (4).

### siRNA knockdown and knockdown verification.

NR9456 cells, BMDMs, and BMDCs were transfected with the indicated siRNAs (9) using Lipofectamine RNAiMAX reagent (Invitrogen). RNA was isolated using the RNeasy minikit (Qiagen). All siRNAs used in this study were previously shown to be on target and to decrease both RNA and protein levels (9). cDNA was made using the SuperScript III first-strand synthesis system for RT-PCR (Invitrogen). RT-PCR was performed using the Power SYBR green PCR master mix kit (Applied Biosystems). Primers for the verification of the knockdowns have been previously described (4, 9).

### IFN-β ELISAs.

BMDMs and BMDCs were transfected with a control- or a Trex1-specific siRNA using Lipofectamine RNAiMAX reagent (Invitrogen). Cells were then infected with MLV^glycoGag^, and the culture medium was harvested 4 hpi. The levels of IFN-β in the culture media were measured using the Legend Max mouse IFN-β ELISA kit (BioLegend) per the manufacturer’s recommendations.

### HIV pseudoviruses.

Retroviral vectors bearing the MMLV Env and HIV (pNL4-3) cores were produced by transient transfection into 293T cells using Lipofectamine 3000 (Invitrogen), as previously described (9). Pseudoviruses were harvested at 48 hpi, and the pseudoviruses were treated with DNase I (20 U/ml for 45 min at 37°C; Roche) and concentrated using Amicon columns.

### Nucleic acid pulldowns.

DDX41myc/his, IFI203-hemagglutinin (HA), and cGAS-V5 plasmids have been previously described (9, 59). 293MCAT cells transfected with pcDNA3.1 (empty vector), cGAS-V5, and DDX41myc/his were infected with virus, and at 4 hpi, the cells were cross-linked with 1% formaldehyde in medium. Cross-linking was quenched with 2.5 M glycine, and extracts were prepared and then incubated overnight with anti-Myc- or anti-HA-agarose beads (Sigma) or anti-V5 antibody (Invitrogen) with G/A-agarose beads (Santa Cruz Biotechnology). The beads were washed with high-salt buffer (25 mM Tris-HCl, pH 7.8, 500 mM NaCl, 1 mM EDTA, 0.1% SDS, 1% Triton X-100, 10% glycerol) and with LiCl buffer (25 mM Tris-HCl, pH 7.8, 250 mM LiCl, 0.5% NP-40, 0.5% sodium deoxycholate, 1 mM EDTA, 10% glycerol). The immunoprecipitated nucleic acid was eluted from the beads at 37°C in 100 mM Tris-HCl, pH 7.8, 10 mM EDTA, 1% SDS for 15 min, and the protein-nucleic acid cross-linking was reversed by overnight incubation at 65°C with 5 M NaCl. The eluted nucleic acid was purified using the DNeasy kit (Qiagen) and analyzed with RT-PCR strong-stop primers (primers P_R_ and P_U5_ in Fig. 3A) or 3′ LTR primers (primers P_3′R_-P_3′L_ in Fig. 4A) (4). For analysis of the tRNA-bound MLV nucleic acid, the same procedure was used, except that the eluted nucleic acid was reverse transcribed prior to PCR with the P_R_ primer and another primer that annealed to nucleotides (nt) 39 to 57 in tRNA^Pro^ (P_tRNA_ in Fig. 3A) (5′-GCTCTCCAGGGCCCAAGTT-3′) (60). For the nuclease treatments, after the nucleic acids were released from the protein cross-link, they were ethanol precipitated and treated at 37°C with 50 U RNase A (Thermo) for 20 min in the presence of 300 mM NaCl, 4 U DNase I (Roche) with the reaction buffer provided with the enzyme for 20 min, or 3 U of RNase H (Thermo) for 20 min in the reaction buffer provided with the enzyme. Samples were digested with proteinase K and phenol-chloroform extracted, and the nucleic acids were subjected to qPCR analysis as described above.

### MLV infection levels.

NR9456 cells were infected with WT or D542N virus, and 2 hpi, cellular DNA and RNA were isolated. RNA was reverse transcribed using a SuperScript III kit (Invitrogen), and the resultant cDNA was used for quantitative PCR using the P_R_-P_tRNA_ primers. DNA was subjected to quantitative PCR using the P_R_-P_U5_ and the P_3′LTRF_-P_3′LTRR_ primers. Bone marrow from C57BL/6 mice was isolated and differentiated to BMDMs and BMDCs. BMDCs and BMDMs were infected with MLV (MOI of 0.1/cell). Cells were harvested at 24 and 48 hpi. For analysis of cell subset infection *in vivo*, newborn mice were infected intraperitoneally (i.p.) with MLV. At 16 dpi, splenocytes were isolated and FACS sorted directly into 15-ml collection tubes using a MoFlo Astrios cell sorter (Beckman Coulter, Inc., Brea, CA) at the UIC Cell Sorting Facility; anti-F4/80-FITC and -CD11c-PE were used to distinguish macrophages and DCs, respectively. DNA was isolated by using the DNeasy kit (Qiagen). Quantitative PCR was performed to measure integrated MLV DNA using the primers 5′-CCTACTGAACATCACTTGGGG-3′/5′-GTTCTCTAGAAACTGCTGAGGGC-3′ and normalized to glyceraldehyde-3-phosphate dehydrogenase (GAPDH).

### Western blot analyses.

Protein extracts from the BMDMs and BMDCs were run on 10% SDS-polyacrylamide gels and transferred to polyvinylidene difluoride (PVDF) Immobilon membranes (Thermo). Rabbit anti-STING, anti-cGAS, anti-phospho-IRF3 (Ser 396), anti-IRF3, anti-TBK1, anti-phospho-TBK1 (Ser 172), and horseradish peroxidase (HRP)-conjugated anti-rabbit antibodies, all from Cell Signaling Technology; mouse monoclonal anti-DDX41 (Santa Cruz Biotechnology); and HRP-conjugated anti-mouse antibody (Sigma-Aldrich) were used for detection, using either ECL Western blot detection reagent or ECL prime Western blot detection reagent (GE Healthcare Life Sciences).

### *In vivo* siRNA knockdown.

siRNAs were purchased from Ambion (Life Technologies, Inc.). The Invivofectamine 3.0 starter kit (Invitrogen Life Technologies, Inc.) was used according to the manufacturer’s protocol. Each siRNA solution (2.5 nmol/µl) was combined with complexation buffer and Invivofectamine reagent for 30 min at 50°C. Footpad injections of the siRNA/Invivofectamine complex or Invivofectamine alone were carried out 48 h prior to infection with MLV (2.5 × 10^5^ infectious center [IC] units/mouse) in the same footpad. Each mouse received 20 nmol of siRNA. After 24 hpi, mice were euthanized and draining lymph node tissues were collected and harvested for RNA isolation. MLV RNA levels were measured by RT-qPCR, as previously described (34). Knockdown of the siRNA-targeted gene was also verified by RT-qPCR as described above.

### *In vivo* infections.

For systemic infections, 2-day-old mice (C57BL/6N, cGAS KO, STING^gt/gt^, and the tissue-specific DDX41 KO mice described in Fig. S7B in the supplemental material) were infected intraperitoneally with 2 × 10^4^ infectious center (IC) units of MLV and then harvested at 18 dpi, and virus titers in spleens were measured by IC assays, as previously described (4). The *in vivo* infection studies were performed at both the University of Pennsylvania and the University of Illinois. The DDX41 knockout mice were housed side by side with the STING^gt/gt^ and cGAS mice and crossed with BL/6N mice from our colony.

### Statistical analysis.

Each experiment was done with 3 technical replicates/experiment. Data shown are the averages from at least 3 independent experiments or as indicated in the figure legends. Statistical analysis for the various experiments was performed using GraphPad Prism software.

### Accession number(s).

All raw data are deposited in the Mendeley data set at https://data.mendeley.com/datasets/j4mgm4v9t3/3.
