# Supplementary material for: DDX41 Recognizes RNA/DNA Retroviral Reverse Transcripts and Is Critical for In Vivo Control of Murine Leukemia Virus Infection
Source: mBio. 2018 Jun 5;9(3):e00923-18. doi: 10.1128/mBio.00923-18 (PMC5989071; doi:10.1128/mBio.00923-18)
Supplement: FIG S1 [file mbo003183913sf1.pdf]

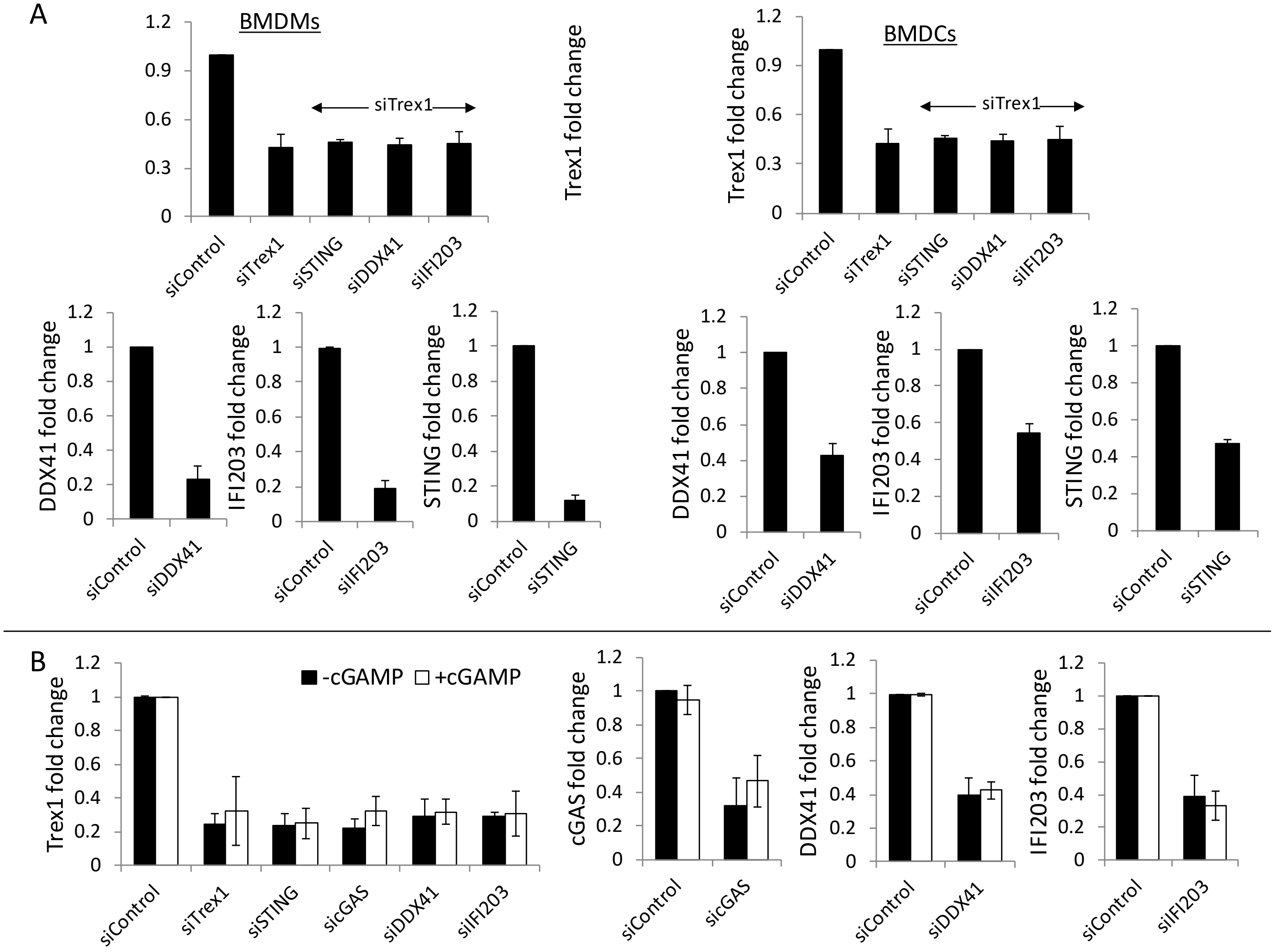

FIG S1 Knockdown verification. Related to Fig. 1. (A) Knockdown of genes in Fig. 1A. The knockdowns of the three target genes (*Ddx41*, *Ifi203*, and *Sting*) were all done in the presence of *Trex1* knockdown, as indicated in the Fig. 1 legend. (B) Knockdown of genes in Fig. 1C. The Fig. 1 legend indicates the details.
