# Supplementary material for: DDX41 Recognizes RNA/DNA Retroviral Reverse Transcripts and Is Critical for In Vivo Control of Murine Leukemia Virus Infection
Source: mBio. 2018 Jun 5;9(3):e00923-18. doi: 10.1128/mBio.00923-18 (PMC5989071; doi:10.1128/mBio.00923-18)
Supplement: FIG S2 [file mbo003183913sf2.pdf]

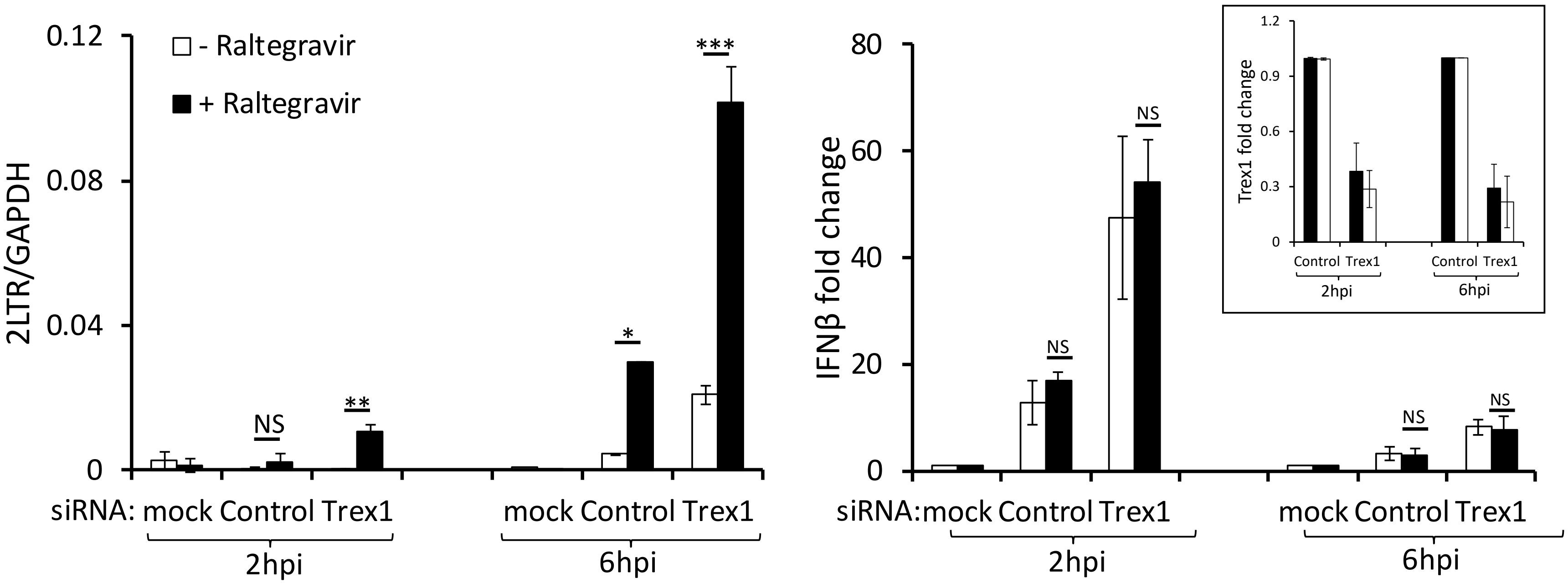

FIG S2 DDX41 senses DNA in the cytoplasm via its DEAD domain. Related to Fig. 2. (A) Increasing nuclear dsDNA by inhibiting proviral DNA integration has no effect on DDX41-mediated sensing. NR9456 cells were pretreated with raltegravir (200 nM) and then infected with MLV (MOI of 2) in the presence of drug. DNA and RNA were isolated from infected cells 2 hpi and analyzed for unintegrated viral DNA (2-LTR) or IFN-β RNA levels. Values are shown as means ± SDs from three experiments. *P* values were determined by an unpaired t test. *, $P \leq 0.05$; **, $P \leq 0.01$; ***, $P \leq 0.001$. The inset shows levels of *Trex1* RNA knockdown.
