# Supplementary material for: DDX41 Recognizes RNA/DNA Retroviral Reverse Transcripts and Is Critical for In Vivo Control of Murine Leukemia Virus Infection
Source: mBio. 2018 Jun 5;9(3):e00923-18. doi: 10.1128/mBio.00923-18 (PMC5989071; doi:10.1128/mBio.00923-18)
Supplement: FIG S3 [file mbo003183913sf3.pdf]

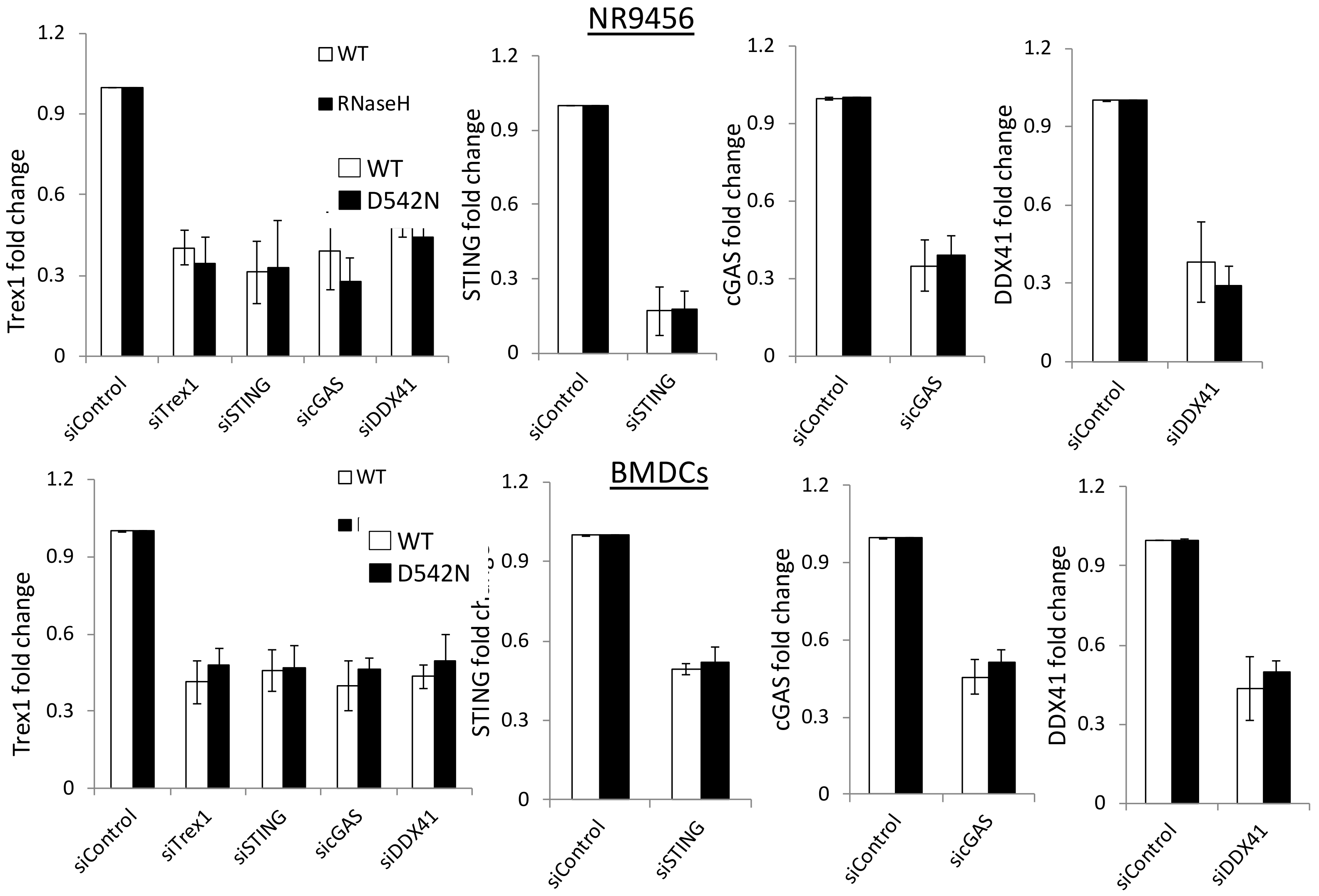

FIG S3. Knockdown verification. Related to Fig. 4. Knockdown of genes in Fig. 4C. The Fig. 4 legend indicates the details.
