# Supplementary material for: DDX41 Recognizes RNA/DNA Retroviral Reverse Transcripts and Is Critical for In Vivo Control of Murine Leukemia Virus Infection
Source: mBio. 2018 Jun 5;9(3):e00923-18. doi: 10.1128/mBio.00923-18 (PMC5989071; doi:10.1128/mBio.00923-18)
Supplement: FIG S4 [file mbo003183913sf4.pdf]

A

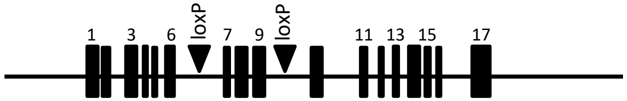

	BMDMs			BMDCs		
**Mouse Strain**	**STING**	**DDX41**	**cGAS**	**STING**	**DDX41**	**cGAS**
C57BL/6	1.243 ± .435	0.731 ± .363	0.654 ± .114	0.666 ± .476	0.530 ± .209	0.170± .02
STING$^{gt/gt}$	0.006 ± .004	0.495 ± .316	0.368 ± .132	0.014 ± .021	0.335 ± .164	0.214 ± .128
cGAS KO	1.294 ± .192	0.670 ± .260	0.001 ± .001	0.65 ± .505	1.10 ± .139*	0.003 ± .003
CD11cCre DDX41$^{fl/fl}$	1.378 ± .778	0.537 ± .267	0.834 ± .661	1.00±.370	0	0.278±.075
LyCre DDX41$^{fl/fl}$	1.237 ± .486	0.003 ± .005	1.407 ± .744	0.89 ± .332	0.469±.172	0.29± .11

	IFNβ/actin (x10^{-3})	
Mouse	BMDM	BMDC
C57BL/6	0.017 ± 0.002	1.1 ± 0.7
STING$^{gt/gt}$	0.015 ± 0.021	ND
cGAS KO	0.016 ± 0.003	0.8 ± 0. 1
LyCre DDX41$^{fl/fl}$	0.022 ± 0.003	ND
CD11cre DDX41$^{fl/fl}$	ND	0. 9 ± 0. 6

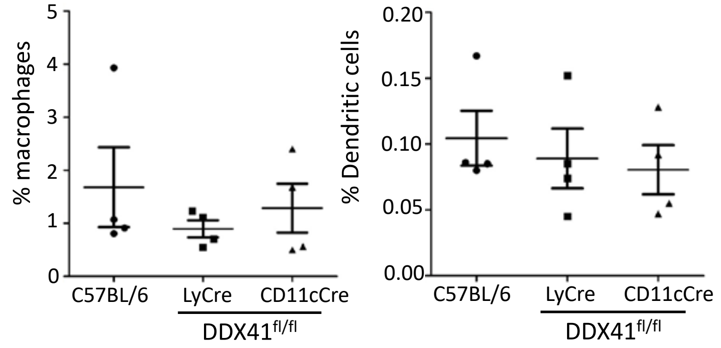

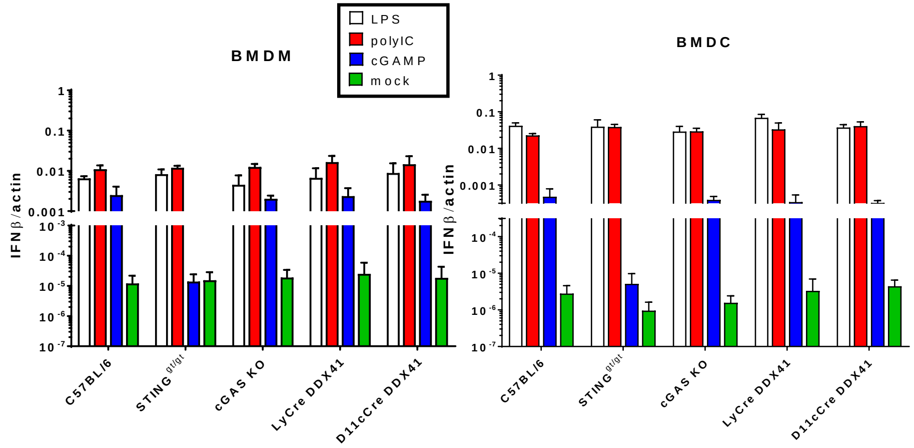

FIG S4 Characterization of *Ddx41* KO mice. Related to Fig. 5 and 6. (A) Mapof the *Ddx41* locus and inserted loxP sites. Expression of Cre recombinase results in the deletion of exons 7 to 9. (B) Quantification of DDX41, cGAS, and STING protein in various knockout mouse cells. Shown are the means ± SDs for 3 independent Western blotting assays of cells from 3 different mice of each strain. *, $P \leq 0.05$ compared to BL/6, *Sting$^{gt/gt}$*, and CD11cCreDDX41 (unpaired t test). (C) Basal IFN-β RNA levels in *Ddx41* KO BMDMs and BMDCs. RNA was isolated from BMDMS of 3 mice and BMDCs of 2 mice each of the indicated genotypes, and qPCR was performed for IFN-β levels, using a standard curve to measure relative levels. Shown are the means ± SDs. Abbreviations: ND, not done. (D) PBMCs from 4 mice of each genotype were stained with conjugated anti-CD11c (DC) or anti-F4/80 (macrophage) antibodies and analyzed by FACS. (E) Treatment of *Ddx41* KO BMDMs and BMDCs with different ligands. BMDMs and BMDCs from the *cGas*, LyCre DDX41 and CD11cCre-DDX41 KO and *Sting$^{gt/gt}$* mice were treated with the indicated ligands, as described in Materials and Methods. RNA was isolated after 6 h of treatment for all ligands and subjected to RT-qPCR. Shown are the averages from 2 experiments (triplicate technical replicates) with cells isolated from different mice.
