# Supplementary material for: DDX41 Recognizes RNA/DNA Retroviral Reverse Transcripts and Is Critical for In Vivo Control of Murine Leukemia Virus Infection
Source: mBio. 2018 Jun 5;9(3):e00923-18. doi: 10.1128/mBio.00923-18 (PMC5989071; doi:10.1128/mBio.00923-18)
Supplement: FIG S5 [file mbo003183913sf5.pdf]

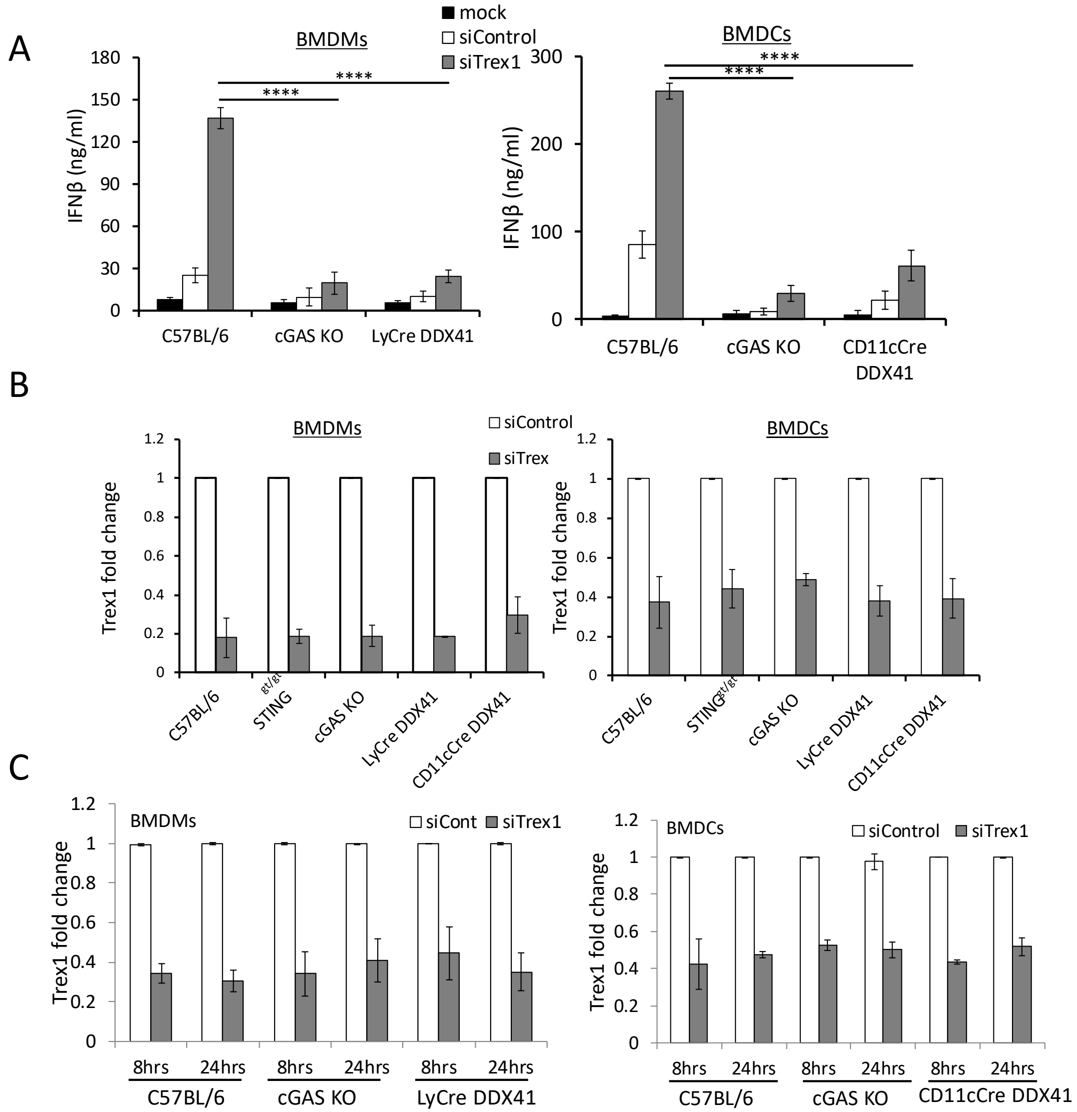

FIG S5 Loss of DDX41 decreases the IFN response to MLV and HIV infection. Related to Fig. 6. A) BMDMs and BMDCs from C57, *cGas* KO, LyCre-DDX41 and CD11cCre-DDX41 mice were treated siCont or siTrex and then infected with $MLV^{glycoGag}$. Four hours later, supernatants were used to perform an ELISA for IFN-β levels. Shown are the average of 3 independent experiments. ****, *P* ≤ 0.0001 (unpaired T-test). B) Verification of Trex1 knockdown in BMDCs and BMDMs in Fig. 6A. C) Verification of the knockdowns in BMDMs and BMDCs in Fig. 6B.
