# Supplementary material for: DDX41 Recognizes RNA/DNA Retroviral Reverse Transcripts and Is Critical for In Vivo Control of Murine Leukemia Virus Infection
Source: mBio. 2018 Jun 5;9(3):e00923-18. doi: 10.1128/mBio.00923-18 (PMC5989071; doi:10.1128/mBio.00923-18)
Supplement: FIG S6 [file mbo003183913sf6.pdf]

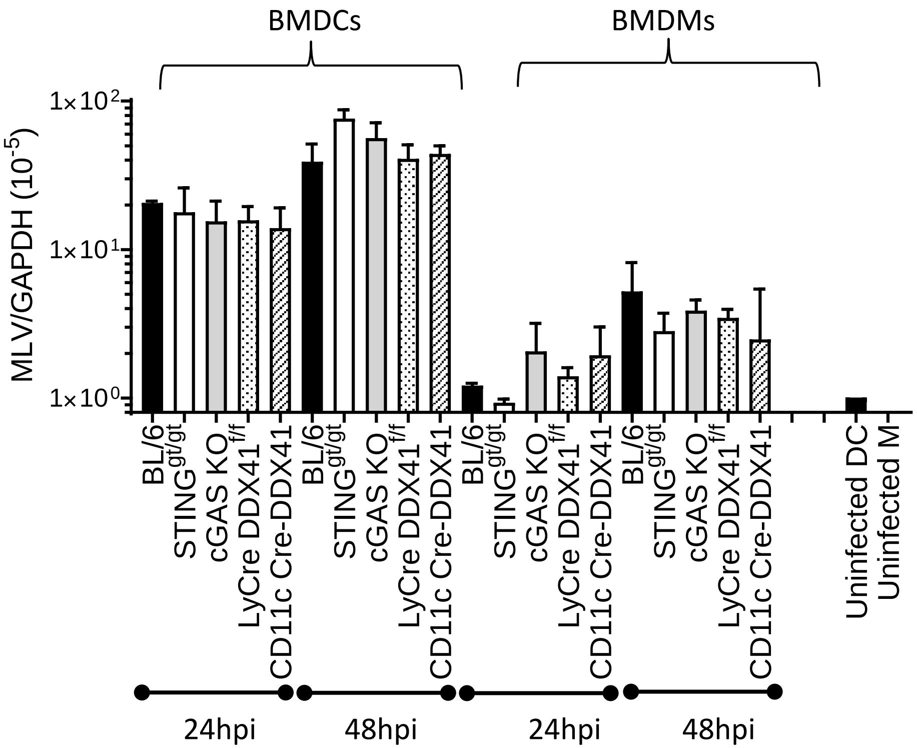

FIG S6 BMDCs are more infected by MLV than BMDMs. Related to Fig. 6. BMDMs and BMDCs from mice of the indicated genotypes were infected with MLV and at 24 and 48 hr pi infection, DNA was isolated from cells and subjected to qPCR, using one primer to mouse genomic DNA and the other to the viral long terminal repeat. Values are shown, as well as means ± standard errors of the means for 2 experiments done for each cell type.
