# Supplementary material for: DDX41 Recognizes RNA/DNA Retroviral Reverse Transcripts and Is Critical for In Vivo Control of Murine Leukemia Virus Infection
Source: mBio. 2018 Jun 5;9(3):e00923-18. doi: 10.1128/mBio.00923-18 (PMC5989071; doi:10.1128/mBio.00923-18)
Supplement: FIG S7 [file mbo003183913sf7.pdf]

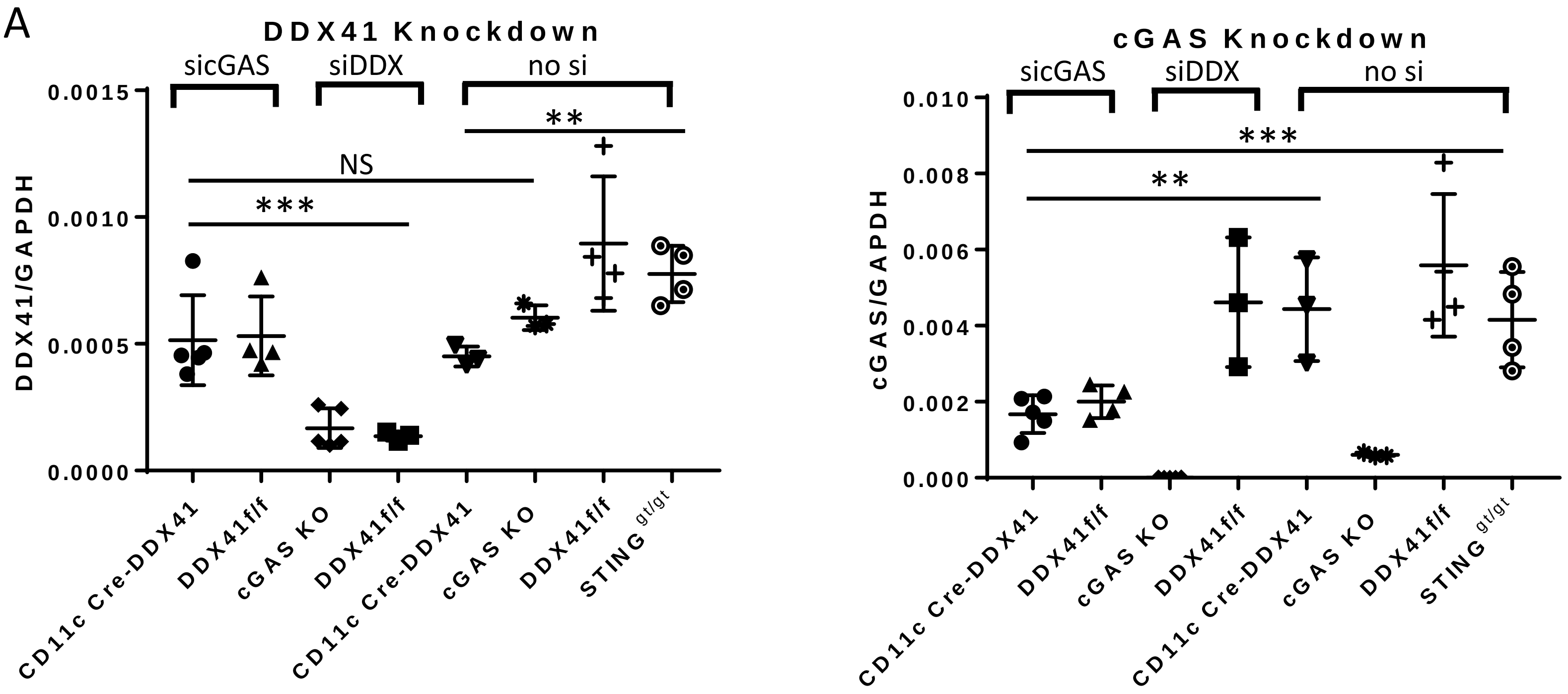

Parental genotypes	Cre+,DDX41+	Cre-,DDX41+	Cre+,DDX41-	Cre-,DDX41-
Cre+,DDX41+	Cre+/+, DDX41+/+	Cre+/-, DDX41+/+	Cre+/+, DDX41+/-	Cre+/-, DDX41+/-
Cre-,DDX41+	Cre+/-, DDX41+/+	Cre-/-, DDX41+/+	Cre+/-, DDX41+/-	Cre-/-, DDX41+/-
Cre+,DDX41-	Cre+/+, DDX41+/-	Cre+/-, DDX41+/-	Cre+/+, DDX41-/-	Cre+/-, DDX41-/-
Cre-,DDX41-	Cre+/-, DDX41+/-	Cre-/-, DDX41+/-	Cre+/-, DDX41-/-	Cre-/-, DDX41-/-

FIG S7 *In vivo* control of retrovirus infection requires both cGAS and DDX41. Related to Fig. 7. A) Knockdown verification of the *in vivo* siRNA experiment presented in Fig. 7A. B) Genotypes of the mice tested for MLV infection in Fig. 7B. Parental mice were $Cre^{+/-}$, $DX41^{+/-}$. Cre refers to either LyCre or CD11cCre, as indicated in the text. Yellow highlighted boxes refer to homozygous, and white boxes to heterozygous, *Ddx41* tissue-specific knockouts; gray boxes refer to WT mice.
